# Universal Visual Features Might Be Necessary for Fluent Reading. A Longitudinal Study of Visual Reading in Braille and Cyrillic Alphabets

**DOI:** 10.3389/fpsyg.2017.00514

**Published:** 2017-04-04

**Authors:** Łukasz Bola, Dominika Radziun, Katarzyna Siuda-Krzywicka, Joanna E. Sowa, Małgorzata Paplińska, Ewa Sumera, Marcin Szwed

**Affiliations:** ^1^Department of Psychology, Jagiellonian UniversityKraków, Poland; ^2^Laboratory of Brain Imaging, Neurobiology Center, Nencki Institute of Experimental BiologyWarsaw, Poland; ^3^CNRS, INSERM U 1127, Institut du Cerveau et de la Moelle Epinière, UMR 7225, Sorbonne Universités – UPMC Univ Paris 06 UMR S 1127Paris, France; ^4^Institute of Pharmacology – Polish Academy of SciencesKraków, Poland; ^5^The Maria Grzegorzewska UniversityWarsaw, Poland; ^6^School for the Blind and Partially Sighted ChildrenKraków, Poland

**Keywords:** reading, visual reading, blindness, vision, learning, human learning, sensory perception

## Abstract

It has been hypothesized that efficient reading is possible because all reading scripts have been matched, through cultural evolution, to the natural capabilities of the visual cortex. This matching has resulted in all scripts being made of line-junctions, such as T, X, or L. Our aim was to test a critical prediction of this hypothesis: visual reading in an atypical script that is devoid of line-junctions (such as the Braille alphabet read visually) should be much less efficient than reading in a “normal” script (e.g., Cyrillic). Using a lexical decision task, we examined Visual Braille reading speed and efficiency in sighted Braille teachers. As a control, we tested learners of a natural visual script, Cyrillic. Both groups participated in a two semester course of either visual Braille or Russian while their reading speed and accuracy was tested at regular intervals. The results show that visual Braille reading is slow, prone to errors and highly serial, even in Braille readers with years of prior reading experience. Although subjects showed some improvements in their visual Braille reading accuracy and speed following the course, the effect of word length on reading speed (typically observed in beginning readers) was remained very sizeable through all testing sessions. These results are in stark contrast to Cyrillic, a natural script, where only 3 months of learning were sufficient to achieve relative proficiency. Taken together, these results suggest that visual features such as line junctions and their combinations might be necessary for efficient reading.

## Introduction

Braille is a tactile alphabet devised by Braille (1829) that has become a standard alphabet for the blind. Although Braille is optimized for tactile reading, sighted teachers and educators rely at work on the visual way of reading Braille. While visual Braille reading has received little attention, it is potentially important for our understanding of visual perception, since learning to read in this script could cause specific challenges. This is because Braille is composed exclusively of dots, which is in stark contrast to all other ‘natural’ scripts. Although they might seem to be very diverse, nevertheless they are made of similar components such as lines, curves and vertices. Frequency distribution of these components matches the visual characteristics of natural scenes that humans encounter in everyday life (Changizi and Shimojo, 2005; Changizi et al., 2006). This suggests that there are some specific, universal features underlying the shape of human visual signs.

**FIGURE 1:**
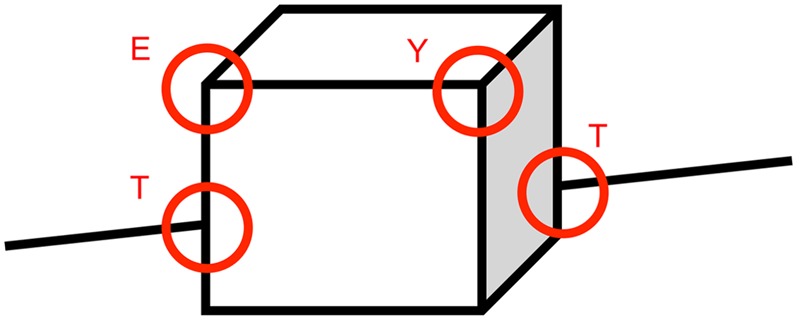
**Natural visual objects have certain invariant properties that are common to most viewpoints.** Object recognition relies heavily on these visual features. The shape T, for instance, is extremely frequent in natural scenes. Whenever one object masks another, their contours almost always form a T-junction. Thus neurons that act as “T-detectors” could help determine which object is in front of which. Reproduced from Dehaene (2009).

Natural visual objects have certain invariant (or non-accidental) properties that are common to most viewpoints. These properties include the manner in which lines meet at vertices to form specific configurations such as T or L, also referred to as line junctions and line coterminations. For example, a table contains several T-junctions where the legs join the table top, and these junctions are common to all but a few unusual viewpoints (**Figure 1**). Object recognition relies heavily on these invariant visual features (Biederman, 1987; Kayaert et al., 2003; Brincat and Connor, 2004; Gibson et al., 2007; Lazareva et al., 2008).

It has been proposed that existing visual scripts are made of similar components (lines, curves, and vertices) because they underwent a “cultural evolution” – matching their visual features to the natural capabilities of the visual cortex (Dehaene and Cohen, 2007; Szwed et al., 2009). According to this view, reading as a cultural invention (invented only about 5400 years ago) takes over parts of neural networks that were shaped for evolutionarily older purposes, for example object recognition. Indeed, the visual word form area (VWFA), part of the left ventral visual stream that underlies identification of visual words, overlaps with regions of ventral visual cortex that are sensitive to the presence of line-junctions and line coterminations that are useful for object recognition (Cohen et al., 2000; Szwed et al., 2011). Thus, it might be that it is not the visual cortex that has adopted for reading, but instead, the writing systems have evolved, by adapting specific visual features, to be easily processed by the human visual system (Dehaene, 2005; Dehaene and Cohen, 2007).

In this paper, we asked how readers process Braille, a script that does not use these features as it is composed exclusively of dots. Proficient visual reading is fast (about 200 words per minute; Carver, 1990) and parallel. In alphabetic writing scripts such as Latin, the effect of word length on reading speed is minimal up to a certain word length, and 8-letter words are read as quickly as 4-letter words. In contrast, first grade children, dyslexic children, and victims of neurological accidents read slowly (about 20 words per minute; Carver, 1990) and letter by letter (Gaillard et al., 2006; Barton et al., 2014). Letter-by-letter reading is also observed when the stimuli are atypical (e.g., pseudowords or rotated words; Weekes, 1997; Cohen et al., 2008). Slow, letter-by-letter reading can thus be used as a measure of reading efficiency.

We used a lexical decision task, a widely used procedure which allows to measure how quickly and how accurately the stimuli are classified as words or pseudowords (Meyer and Schvaneveldt, 1971), with stimuli of different letter length, to examine the reading speed, accuracy and the world length effect in a group of adults learning to read Braille visually. As a control group, we chose participants of a Russian language course, and thus Cyrillic – a ‘natural’ visual script – learners. Russian was chosen since it is the most popular second language in Poland which requires learning new alphabet (Polish is written in Latin alphabet). Our objective was to test a critical prediction of the “neuronal recycling” hypothesis: learning to read Braille visually should be much slower and less efficient than learning to read in regular visual scripts.

## Materials and Methods

### Subjects

Thirty-six individuals initially took part in the visual Braille part of study (32 females, 4 males; median age = 27, range = 22–49). Simultaneously, they took part in a tactile Braille course. Their MRI imaging results and the exact process of their tactile braille learning have been reported elsewhere (Bola et al., 2016; Siuda-Krzywicka et al., 2016, respectively). All were right-handed, fluent in Polish and had normal or corrected to normal vision. They were either Braille teachers/educators (16 subjects), special education studies students, specializing in blindness and related disabilities (15 subjects) or close relatives of blind people (five subjects). Thirty-three participants knew how to read visually presented Braille from the beginning of the study (at least 60% level of accuracy in the visual Braille lexical decision task). Due to variety of reasons (lack of time, personal reasons, loss of interest) seven subjects resigned from the study. Twenty-nine subjects completed the course (26 females, 3 males; median age = 27, range = 22–49). Among the latter, two subjects did not attend some testing sessions.

In the Cyrillic group, 47 subjects were recruited for the experiment (36 females, 11 males; median age = 21, range = 20–27). All of them were attending Russian course and thus learning Cyrillic. Twenty-nine of them decided not to continue their course due to loss of interest in Russian or lack of time to attend the course, therefore they were not able to participate in subsequent parts of the study. Eighteen subjects took part in all sessions (14 females, 4 males; median age = 21, range = 20–25). Seventeen of them were right-handed, all fluent in Polish and had normal or corrected to normal vision. The difference in age between the groups was significant [*t*(41) = 3.802, *p* < 0.001]. The primary limiting factor was the scarcity of subjects willing to learn Braille. Nevertheless, despite this age difference as well as differences in the way the two courses were designed, we argue that the comparison between Braille and Cyrillic groups remains valid (see Discussion).

The experiment was approved by the local ethical committee (Komisja ds. Etyki Badań Naukowych) at the Jagiellonian University, Kraków. A written consent was obtained from each participant.

### Outline of the Study and the Braille Course

**FIGURE 2:**
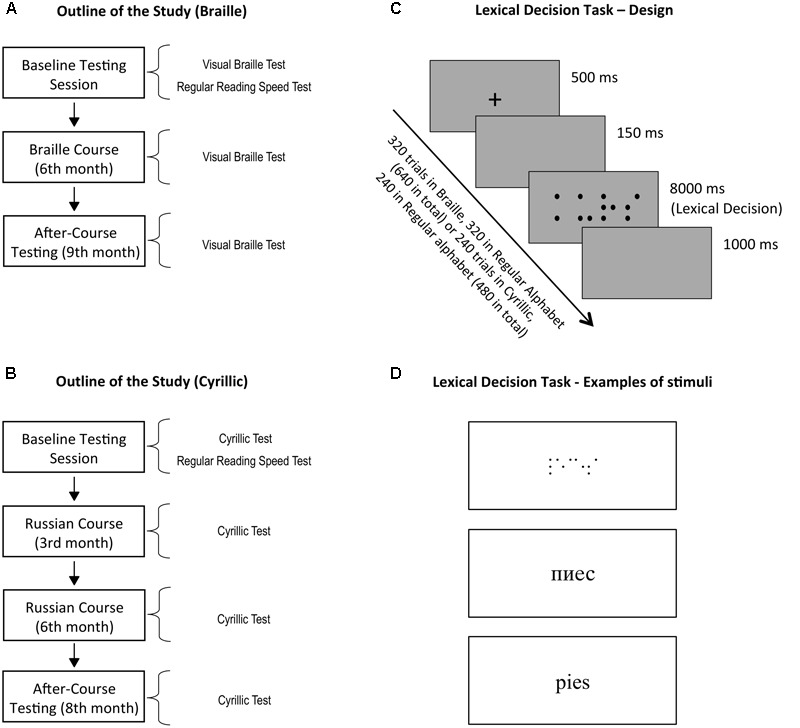
**Outline of the study and experimental procedures. (A)** Braille course participants were tested three times: during the baseline testing session and after 6 and 9 months of the course. **(B)** Russian course participants were tested four times: during the baseline testing session and after 3, 6, and 8 months of the course. **(C)** In each testing session, subjects performed a lexical decision task, in which subjects were asked to visually read items appearing on the screen and make a word/pseudoword decision. Braille course participants performed this task in Braille and Latin (i.e., native) alphabets. Russian course participants performed it in Cyrillic and Latin. **(D)** Examples of stimuli in Braille, Cyrillic, and Latin alphabets.

The outline of the study is shown in **Figures 2A,B**. The Braille course lasted 9 months. It included some pre-existing material from Polish (Paplińska, 2009) and English textbooks, yet it created a new, original curriculum, tailored to the needs of sighted people. The course was designed by two experienced Braille teachers (MP and ES, co-authors of this article). The Russian course also lasted 9 months and included basic textbooks for Russian at A1 (beginner) level (e.g., Granatowska and Danecka, 2002).

The Braille course relied primarily on participants’ individual work. Every month they were given 30 exercises, each on a single sheet. They were asked to complete one exercise per day. Each exercise was first completed tactually and then checked visually. Thus, the subjects received approximately the same amount of tactile and visual Braille training. The daily workload, as reported by the subjects, varied from 10 to 30 min. Once a month a group meeting was organized, during which participants were given a subsequent set of Braille exercises and could resolve potential concerns with an expert Braille teacher. Russian learners attended their course once a week. Each lesson took 90 min. Their 9-month workload was assessed by Jagiellonian Language Center as 6 ECTS, which equals 150–180 h.

### Visual Braille/Cyrillic Reading Test

A lexical decision task was used to measure the speed and accuracy of visual Braille and Cyrillic reading. Subjects from the Braille group were instructed to visually read letter strings appearing on the screen either in Braille or in native (Latin) alphabet, and decide whether they formed a valid Polish word (**Figure 2C**). They completed the task three times – at the beginning of the Braille course, in its 6th month and at its end, i.e., the 9th month. Subjects from the Cyrillic group performed the same task, but on Polish words and pseudowords transliterated to Cyrillic, and in their native Latin alphabet. They performed the task before their first Russian lesson, after 3, 6, and 8 months of the course.

The stimuli in the Braille group consisted of 640 items (320 Polish words and 320 pseudowords). 80 words and 80 pseudowords were 4-letters and 1 syllable long, 160 words and 160 pseudowords were 6-letters and 2 syllables long, and 80 words and 80 pseudowords were 8-letters and 3 syllables long. The stimuli in the Cyrillic group were a minimally shortened (due to time constraints) version of the Braille stimuli and included 480 items (240 Polish words and 240 pseudowords), where 60 words and 60 pseudowords were 4-letters and 1 syllable long, 120 words and 120 pseudowords were 6-letters and 2 syllables long, and 60 words and 60 pseudowords were 8-letters and 3 syllables long. All words were of low-to-moderate frequency (from 1 to 10 occurrences per million) and the mean frequency was matched across the groups of words of different lengths. The neighborhood size, as defined by the OLD20 measure (Yarkoni et al., 2008), was equated between words and pseudowords of the same length. Full equation of neighborhood size across items with different lengths was impossible in the Polish language. Therefore, we decided to introduce two levels of the neighborhood size. 4-letter items were matched in the neighborhood size with half (i.e., 80 words and 80 pseudowords) of the 6-letter items. The second half of the 6-letter items was equated in the neighborhood size with the 8-letter items. The SUBTLEX-PL database was used to obtain the psycholinguistic characteristics of the items (Mandera et al., 2015). For each item length, half of both words and pseudowords were displayed in Braille/Cyrillic, and the second half of the items were displayed in the subjects’ native alphabet. The Braille/Cyrillic vs. native alphabet conditions were counterbalanced between participants.

To make the font used for the Braille stimuli as similar to the natural visual alphabet as possible, we used Visual Braille, a TrueType font displaying only the “raised” dots without empty portions as a reference (**Figure 2D**). In case of the Cyrillic alphabet, the font used was MAC C Times, a typical font used by Cyrillic readers. For the native alphabet condition it was Times New Roman. The font size of all types of stimuli was 20.

### Data Analysis

Two subjects from the Braille group were excluded due to missing data from either the second or third testing session. In the reaction time (RT) analysis, another two subjects were excluded, as their performance in Braille reading in the first testing session was at the chance level (accuracy = 49.1 and 49.7%). Thus, the final accuracy analysis was performed on 27 subjects, and the RT analysis was performed on 25 subjects. In case of the Cyrillic group, 18 subjects attended all parts of the study. Given that their average performance in Cyrillic reading in the first testing session was at chance level (mean accuracy = 50.4%, CI95% = 49.3–51.4%), RT data from this session were discarded.

Due to sphericity violations in RT data (Mauchly’s sphericity tests for session and item length factors – all *p* < 0.001), we decided to use MANOVA instead of a classical ANOVA, since the former is free of sphericity assumptions. According to statistical guidelines, MANOVA should be used instead of a repeated-measures ANOVA when assumptions of sphericity are violated (e.g., O’Brien and Kister Kaiser, 1985). Because of different length of lexical decision task experiment in each group, the RT data were analyzed in two steps. First, within-group comparisons were performed separately for each group using 3 × 3 × 2 MANOVA with Session (first, second, third in the Braille group and second, third, fourth in the Cyrillic group), Item Length (4-, 6-, 8-letters) and Item Type (word, pseudoword) as within-subject factors. These within-group MANOVAs included all items in a non-native alphabet that were presented to a given group (i.e., 320 Braille items in the Braille group and 240 Cyrillic items in the Cyrillic group). Then, between-group comparisons were performed using a 3 × 3 × 2 × 2 MANOVA with Group as a between-subject factor. This MANOVA included the same number of items for each group (i.e., 240 Braille items for the Braille group and 240 Cyrillic items for the Cyrillic group). This procedure allowed us to avoid losing statistical power in within-group comparisons and, at the same time, to perform unbiased between-group comparisons. All RT data analyses were performed on medians, which are more robust, i.e., less sensitive to outliers than means. The MANOVA approach was also used to analyze between-session differences in accuracy in each subject group (Mauchly’s sphericity tests for the session factor – all *p* < 0.001).

## Results

### Reading Speed and Length Effect in Visual Braille

**FIGURE 3:**
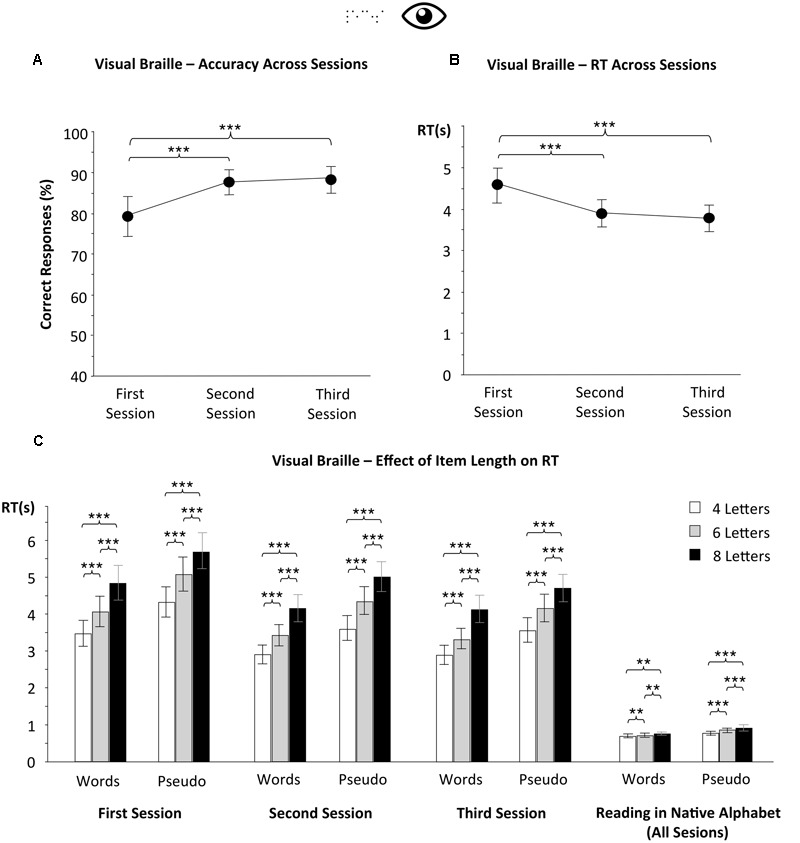
**Visual Braille reading.** Although subjects progressed in their visual Braille reading, the process remained very slow and serial. During the course, we observed **(A)** an increase in mean accuracy as well as **(B)** a decrease in reaction time (RT) when subjects performed the Braille lexical decision task. However, **(C)** Braille reading remained very slow when compared with reading a regular alphabet. The item length was virtually constant through all testing sessions, both for words and pseudowords. Error bars represent 95% confidence intervals. Asterisks indicate a significant difference between the results of specific testing sessions (^∗∗∗^*p* < 0.001, ^∗∗^*p* < 0.01).

The overall response accuracy for Braille in the first testing session was 78.9% (**Figure 3A**; CI95% = 73.6–84.2%). This confirms that at the onset of the course, most subjects already had considerable expertise in visual Braille reading. However, their Braille reading was very slow (**Figure 3B**; mean RT = 4587 ms, CI95% = 4168–5007 ms) when compared with their reading of the native alphabet (mean RT = 862 ms, CI95% = 781–943 ms; see also **Figure 3C**). In that first testing session, we also observed an item length effect, i.e., we saw that the reading speed decreased with item length (all *p* < 0.001). This effect was very pronounced and similar for words and pseudowords – the RT for 8-letter items was 1200–1300 ms higher than for items of 4-letter length (**Figure 3C**). Thus, each Braille letter added an additional 300 ms to RT. This is in a clear contrast to reading in the native script (Latin), in which the length effect for words was minimal (63 ms difference between 4- and 8-letter words; **Figure 3C**).

**FIGURE 4:**
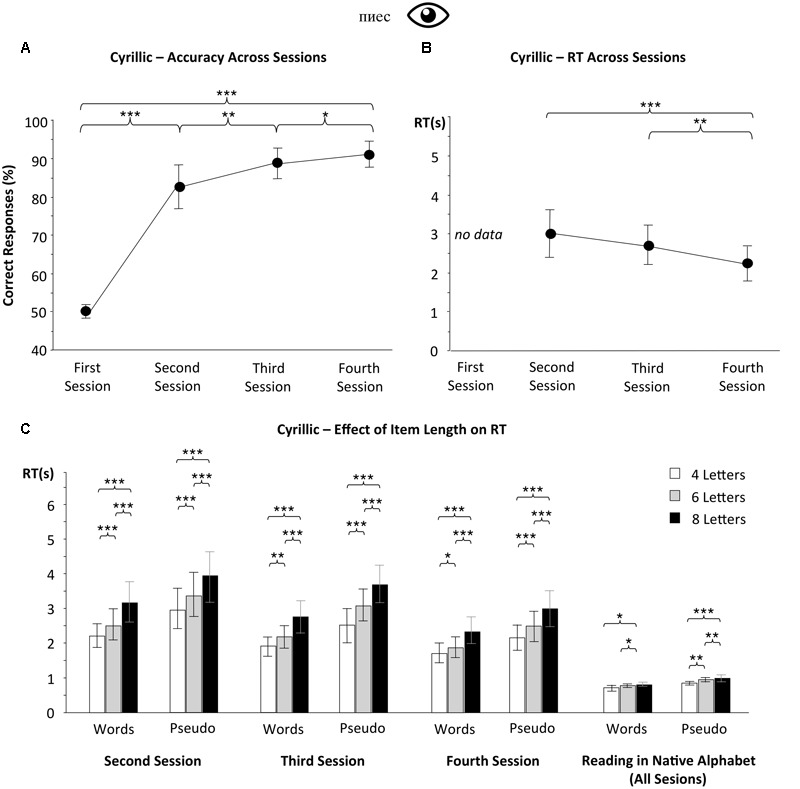
**Cyrillic alphabet reading.** Contrary to visual Braille, the Cyrillic alphabet can be quickly mastered to a high level of fluency. The item length effect is smaller for words than for pseudowords and is modulated by readers’ expertise. A mere 3 months of Cyrillic learning was sufficient to **(A)** progress in the lexical decision task from chance level to accuracy around 85%. Also **(B)** RT decreased to a relatively low level. After 6 and 8 months of course, the item length effect **(C)** for words was small. Notably, even after only 3 months of learning Russian, the item length effect both for words and pseudowords was smaller than in the case of the Braille learners. Error bars represent 95% confidence intervals. Asterisks indicate a significant difference between the results of specific testing sessions (^∗∗∗^*p* < 0.001, ^∗∗^*p* < 0.01, ^∗^*p* < 0.05). RT data from the first testing session were discarded because subjects performed the word/pseudoword discrimination at chance level.

As the course progressed, we observed an increase in accuracy (**Figure 3A**; mean = 88%, CI95% = 84.3–91.7% in the 9th month) and speed (**Figure 3B**; mean = 3800 ms, CI95% = 3468–4132 ms in the 9th month) of visual Braille reading [effect of testing session: *F*(2,25) = 26.026, *p* < 0.001; *F*(2,23) = 24.525, *p* < 0.001, respectively]. Still, even after 9 months of training, the reading speed for Braille was much slower than for Cyrilic, a “normal” visual script (see below). The effect of item length on word reading speed remained virtually constant across all testing sessions (**Figure 3C**), despite subjects’ gains in Braille reading expertise (10% increase in accuracy, 675 ms increase in speed – see **Figures 3A,B**) – there was 1247 ms difference in RTs between 4- and 8-letter words in the last session (CI95% = 1076–1418 ms; **Figure 4C**), whilst in the first testing session it was 1387 ms [CI95% = 1178–1597 ms; difference not significant – *t*(24) = 1.397, *p* = 0.175]. Significantly, MANOVA on both visual Braille and Cyrillic subject groups (3 × 3 × 2 × 2, see Materials and Methods) revealed that even if one took into account the residual difference in reading speed between these two alphabets [main effect of group – *F*(1,41) = 24.7, *p* < 0.001], visual Braille readers still had a much larger word length compared to Cyrillic readers [interaction group^∗^item length – *F*(2,40) = 7.552, *p* = 0.002].

### Reading Speed and Length Effect in Cyrillic

During the baseline session (before the first Russian lesson) subjects performed the lexical decision task at chance level (mean accuracy = 50.4%, CI95% = 49.3–51.5%). This showed that they were indeed naïve in Cyrillic, and that the visual similarity between Cyrillic and Latin alphabets was not sufficient to read Cyrillic words, even very slowly. After the first 3 months of the course, the mean accuracy increased to 83.9% (CI95% = 80–87.8%; **Figure 4A**) with mean RT of 3281 ms (CI95% = 2766–3797 ms, **Figure 4B**). This means that even beginner Cyrillic readers (**Figure 4A**) reached a better reading performance than experienced Braille readers before their additional Braille training (**Figure 3A**). Similar to the effect in Braille readers, we observed a significant item length effect on reading speed, both for words and pseudowords (all *p* < 0.05) – where reading speed decreased with item length (**Figure 4C**). The RT for 8-letter items was 1099 ms higher than for items of 4-letter length (**Figure 4C**).

After 6 and 8 months of learning, the subjects’ accuracy increased further (**Figure 4A**; 88.9%, CI95% = 85.4–92.3 and 91.3%, CI95% = 88.3–94.2%, respectively). Mean RTs decreased as well [**Figure 4B**; mean = 2637 ms, CI95% = 2221–3052 and 2280 ms, CI95% = 1887–2673 ms, respectively; effect of testing session: *F*(2,16) = 18.591, *p* < 0.001; *F*(2,16) = 27.558, *p* < 0.001, respectively]. Moreover, item length effect for words also decreased significantly – there was only an 640 ms difference in RTs between 4- and 8-letter words in the last session (CI95% = 475–806 ms; **Figure 4C**), whilst in the first testing session it was 1006 ms [CI95% = 739–1274 ms; difference significant – *t*(17) = 3.514, *p* = 0.003].

### Reading in Native Alphabet (Latin Alphabet)

Finally, as a control of linguistic material in our task, we performed an accuracy analysis on native alphabet items in both Braille and Cyrillic group. The overall responses’ accuracy in Braille group for the first, second, and third session was 97.7, 97.3, and 97.2%, respectively, whereas overall RT was 862, 812, and 806 ms, respectively. In the Cyrillic group, the overall responses’ accuracy for the first, second, third, and fourth session was 97.4, 98.1, 98.1, and 98.2%, respectively, whereas overall RT was 950, 867, 815, and 789 ms, respectively.

Furthermore, subjects performed a standard test of visual reading speed in the subject’s native alphabet (Latin), to relate their typical reading speed to their Braille performance. The passage from a Polish book *Farsa Panny Heni* by Maria Rodziewiczówna was used. It consisted of 400 words. Subjects were asked to read the text silently, as fast and carefully as possible, and press a button right after they finish. Next, a paper-pencil test was administered to verify the passage comprehension. The test included 10 multiple-choice questions with three answer option. Total reading time in the reading speed test and accuracy in the comprehension test were collected as dependent measures. The mean reading speed for the native alphabet was 209 words per minute (WPM) in the Braille group (CI95% = 190–227 WPM) and 217 WPM in the Cyrillic group (CI95% = 178–257 WPM). These values can be qualified as a standard adult visual reading speed (Hunziker, 2006). There was no significant difference in reading speed between both groups [*t*(42) = 0.479, *p* = 0.63]. We found a correlation between the speed of reading in the native alphabet and RT for Braille reading in the final testing session, meaning that fast regular readers were also fast visual Braille readers (*r* = -0.52, *p* = 0.009]. The correlation between reading speed in the native alphabet and Cyrillic reading speed in the final testing session was not significant (*r* = 0.012, *p* > 0.05).

## Discussion

In this experiment, we studied visual reading in Braille and in a typical visual script – Cyrillic. We observed that visual Braille reading remains very slow, even after extensive training. Braille learners that had significant prior experience in Braille read slower than Cyrillic learners after only 3 months of an introductory course in Russian. Also, the word length effect – the increase of RT per each additional letter, a hallmark of serial letter-by-letter reading, was longer for Braille learners as well.

Due to scarcity of subjects willing to learn Braille, and the particular constraints of recruiting Cyrillic subjects in a university language course, the two groups were not ideally matched. However, nearly all differences between groups: previous knowledge of visual Braille vs. lack of any Cyrillic training before the start of the Russian course; the different daily workload – visual Braille training each day vs. once-per-week Russian classes and the fact that the Braille group also studied tactile Braille, with possible synergistic effects on visual Braille learning, were in favor of the Braille group. Furthermore, the last session in Cyrillic group took place after 8 months of the course, whilst in the Braille group it took place 1 month later. Nevertheless, the results of the Cyrillic group were always significantly better, both in terms of accuracy and RT.

It has been proposed that fluent reading is possible due to object-related characteristics of visual scripts, such as high-contrast line-junctions. Changizi and Shimojo (2005) and Changizi et al. (2006) have shown that visual signs have been selected to match the configuration distribution ranks found in natural scenes. In line with these findings, Szwed et al. (2009) have observed that when the line-junctions are preserved in pictures of objects and printed words, participants made fewer errors and were faster to respond in a naming task relative to preserved line midsegments, which suggests that vertex invariants are used for object recognition (see also: Lanthier et al., 2009; Rosa et al., 2016). The importance of line-junctions for letter recognition has also been documented by Fiset et al. (2008, 2009) using the bubbles technique that is free from *a priori* assumptions about relative importance of different visual features.

The “neuronal recycling” hypothesis (Dehaene and Cohen, 2007) takes these observations as an evidence that reading scripts match the natural capabilities of the visual system. According to this hypothesis, cultural inventions (e.g., reading, arithmetic) take over parts of neural networks that were shaped for evolutionarily older purposes. Due to plasticity of those recruited areas, newly acquired skills are effectively supported, yet they are still constrained by their evolutionary origins. To be effective, cultural inventions thus have to match the natural capabilities of “recycled” areas. In support of this hypothesis, it was shown that the reading region in the brain, VWFA (Cohen et al., 2000; Dehaene and Cohen, 2011; but see also Price and Devlin, 2011), develops from the object recognition region in the ventral steam (Dehaene et al., 2010), and that it overlaps specifically with the part of the object recognition area that is sensitive to the presence of line-junctions (Szwed et al., 2011; its location is also determined by connections with language areas – see Saygin et al., 2016).

Our results show that Braille – a script not adapted to capabilities of the visual cortex – is processed in a very slow and serial fashion, even in subjects with prior visual Braille experience and their skills’ improvement during the course. Visual Braille reading is very arduous – although our Braille readers had considerable prior experience with this script, it took them 9 months to progress from 79 to 88% accuracy and their reading remained very slow, even comparing to beginners in a ‘natural’ script, Cyrillic. The latter progressed from chance level to accuracy above 80% in the lexical decision task in only 3 months. After 6 and 8 months, their accuracy approached 90% (88 and 91%, respectively).

Item length effects were also different in visual Braille and Cyrillic. In visual Braille reading, it was large and similar for pseudowords and words – every additional letter increased the RT by 300 ms. Despite the 680 ms drop in mean RT between the first and last session, the item length effect remained for Braille constant. Such slow, serial reading is reminiscent of reading atypical stimuli – for example rotated words (Cohen et al., 2008). It can also be found in patients with pure alexia (e.g., Gaillard et al., 2006; mean RT = 2240 ms). Nevertheless, even compared with those cases, visual Braille reading seems to be very slow (mean RT after the course = 3800 ms). In Cyrillic, the item length effect was noticeably smaller: there was an 640 ms difference in RTs between 4- and 8-letter words in the last session, whilst in the first testing session it was 1006 ms. Taken together, these results mean that Cyrillic can be quickly mastered to a relatively high level of fluency.

It is possible that with additional intensive training, one could reach a higher visual Braille reading efficiency. However, our results show that efficient reading is much harder (or maybe even impossible) to achieve for visual Braille than for a typical visual script. The slow and serial character of reading visual Braille – a script that lacks the universal visual features common to all other known scripts – suggest that those specific features might be necessary for fluent reading.

## Ethics Statement

This study was carried out in accordance with the recommendations of Committee for Research Ethics of the Department of Psychology of the Jagiellonian University with written informed consent from all subjects. All subjects gave written informed consent in accordance with the Declaration of Helsinki. The protocol was approved by the Committee for Research Ethics of the Institute of Psychology of the Jagiellonian University.

## Author Contributions

Conceived and designed the experiment: ŁB, KS-K, MS. Performed the experiment and analyzed the data: DR, ŁB, KS-K. Piloted the experiment: JS. Designed and taught the Braille course: MP, ES. Wrote the paper: DR, ŁB, KS-K, MS.

## Conflict of Interest Statement

The authors declare that the research was conducted in the absence of any commercial or financial relationships that could be construed as a potential conflict of interest. The reviewer BH and the handling Editor declared their shared affiliation, and the handling Editor states that the process nevertheless met the standards of a fair and objective review.
